# Excision of DNA fragments with the *piggyBac* system in *Chrysanthemum morifolium*

**DOI:** 10.5511/plantbiotechnology.23.0324a

**Published:** 2023-06-25

**Authors:** Mitsuko Kishi-Kaboshi, Ayako Nishizawa-Yokoi, Ichiro Mitsuhara, Seiichi Toki, Katsutomo Sasaki

**Affiliations:** 1 Institute of Vegetable and Floriculture Science, National Agriculture and Food Research Organization, 2-1 Fujimoto, Tsukuba, Ibaraki 305-0852, Japan; 2 Institute of Agrobiological Sciences, National Agriculture and Food Research Organization, 3-1-3 Kannondai, Tsukuba, Ibaraki 305-8604, Japan; 3 Institute of Agrobiological Sciences, National Agriculture and Food Research Organization, 2-1-2 Kannondai, Tsukuba, Ibaraki 305-8602, Japan; 4 Laboratory of Plant Genome Engineering, Department of Plant Life Science, Faculty of Agriculture, Ryukoku University, 1-5 Yokotani, Seta Oe-cho, Otsu, Shiga 520-2194, Japan

**Keywords:** *Chrysanthemum morifolium*, DNA excision, genome engineering, *piggyBac*, transposon

## Abstract

*Chrysanthemum morifolium* is one of the most popular ornamental plants in the world. However, as *C. morifolium* is a segmental hexaploid, self-incompatible, and has a sizable heterologous genome, it is difficult to modify its trait systematically. Genome editing technology is one of the attractive methods for modifying traits systematically. For the commercial use of genetically modified *C. morifolium*, rigorous stabilization of its quality is essential. This trait stability can be achieved by avoiding further genome modification after suitable trait modification by genome editing. Since *C. morifolium* is a vegetatively propagated plant, an approach for removing genome editing tools is required. In this study, we attempted to use the *piggyBac* transposon system to remove specific DNA sequences from the *C. morifolium* genome. Using the luminescence as a visible marker, we demonstrated that inoculation of *Agrobacterium* harboring hyperactive *piggyBac* transposase removes inserted 2.6 kb DNA, which harbors *piggyBac* recognition sequences, from the modified Eluc sequence.

## Introduction

*Chrysanthemum morifolium* Ramat. is the most economically valuable flower worldwide. It is mainly a hexaploid (2n=6×=54) with the loss or gain of several chromosomes (Shibata and Kawata 1986). Genome size of *C. morifolium* would be fluctuated between cultivars and generally considered to have a large genome (12.3–24.7 Gbp, https://www.asteraceaegenomesize.com/# (Accessed Oct 17, 2021)). The recent study using flow cytometry showed that one of the *C. morifolium* cultivar, ‘Sei-Marin,’ have 7.93 Gb genome (Nakano et al. 2021). The origin of *C. morifolium* is also complicated, and more than six wild *Chrysanthemum* species are suggested to be involved in the origin of *C. morifolium* (Ma et al. 2020). Additionally, most *C. morifolium* cultivars are self-incompatible (Wang et al. 2014), sexually reproduced for breeding, and propagated asexually for commercial production. Therefore, using genetic markers for breeding a broad range of traits is quite challenging, and the production of cultivars is expensive. Transgenic technology is attractive for adding novel traits to *C. morifolium*. Several interesting transgenic *C. morifolium* have been developed, with traits such as insect resistance (Shinoyama et al. 2002) and naturally impossible violet and blue flower colors (Noda et al. 2013, 2017). For the commercial use of transgenic *C. morifolium*, it is legally necessary to confer sterility to these plants to avoid gene transfer in the area where it can pollinate wild species (Aida et al. 2018, 2020). Genome editing is one of the potential methods to systematically modify traits, such as sterility. We have previously demonstrated the application of the clustered regularly interspaced short palindromic repeats (CRISPR)-associated protein 9 (Cas9) system in *C. morifolium* (Kishi-Kaboshi et al. 2017). Recently, Shinoyama et al. (2020) successfully knocked out *DMC1* by genome editing and conferred male and female sterility in *C. morifolium*, which will be essential for cultivating transgenic *C. morifolium* in the open field under guidelines and directives of the Cartagena Protocol on Biosafety. The next step will be to create commercially available genetically modified *C. morifolium* that is incapable of exogeneous gene transfer. After the intended traits have been developed, it is necessary to restrict the nuclease activity used for genome editing, such as *Cas9* or *TALEN*, as residual enzyme activity has a risk of unintended genome editing on off-target sites during a long vegetative propagation period. The removal of these exogeneous genes for genome editing enzymes from genome-edited *C. morifolium* is one of a possible method to avoid the risk of unintended genome editing, including off-target mutation. Therefore, we used the *piggyBac* transposon system to excise unnecessary DNA sequences from the *C. morifolium* genome. To the best of our knowledge, this study is the first to undertake such a task.

DNA transposons are genetic elements that can move from one location to another in the host genome. The *piggyBac* transposon was isolated from the cabbage looper moth *Trichoplusia ni* (Cary et al. 1989). It is unique because it rarely leaves a trace of transposon insertion on the host genome (Fraser et al. 1996). Hyperactive *piggyBac* transposase (hyPBase) has a 17-fold excision rate from that of the original *piggyBac* transposase, and its frequency of footprint occurrence is low (approximately 1%) (Yusa et al. 2011). In plant, the *piggyBac* system with *hyPBase* was first used to rice (Nishizawa-Yokoi et al. 2014). We used emerald luciferase (Eluc) luminescence to monitor DNA excision from transgene-containing transposon recognition sites to evaluate whether the *piggyBac* transposon system works in *C. morifolium*.

## Materials and methods

### Vector construction

The pBI121 vector was used as the backbone in this report. The pBI121 vector was digested with *Hind*III and *Eco*RI, and assembled with PCR products using Gibson Assembly Master Mix (NEB, https://www.nebj.jp/).

**Figure 1:**
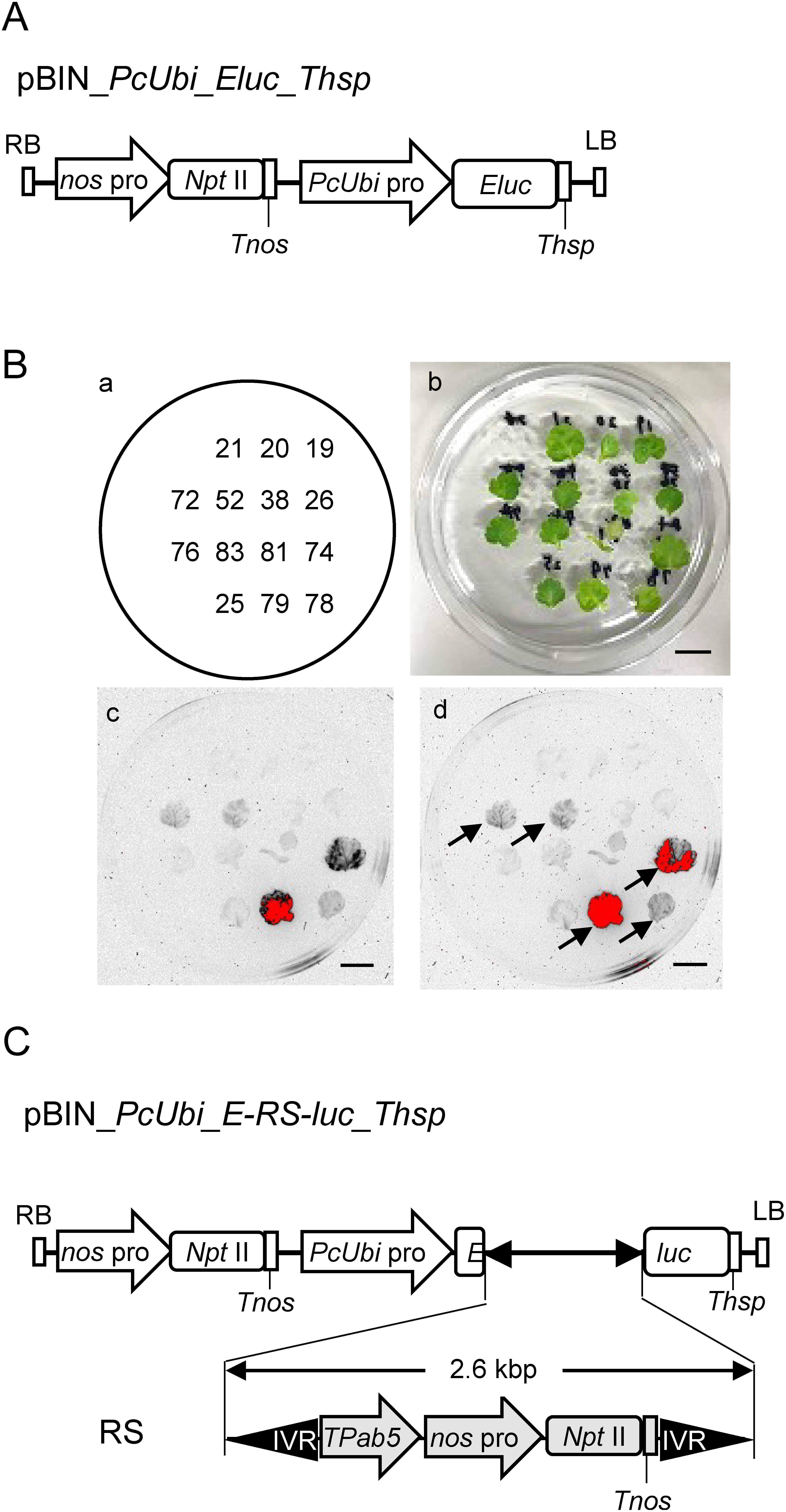
Figure 1. The generation of Eluc and E_RS_luc transgenic *C. morifolium* plants. A: Structures of pBIN_*PcUbi_Eluc_Thsp* to generate transgenic Eluc plants. B: The location of each transgenic leaf, with its line numbers, is shown in the left upper panel (a). A photograph of the leaves is shown in the right upper panel (b). Luminescence in leaves of transgenic Eluc plant ((c) left bottom, 20 min exposure; (d) right bottom, 120 min exposure). Leaves from independent transgenic Eluc plants were treated with D-luciferin. Bars indicate 1 cm length. C: Structures of pBIN_*PcUbi_E-RS-luc_Thsp* to generate transgenic E_RS_luc plants. *Eluc*, *emerald luciferase*; IVR, inverted repeat of *piggyBac* transposon; LB, left border; *nos* pro, *nos* promoter, *npt* II; *neomycin phosphotransferase* for kanamycin resistance, *PcUbi*, *ubiquitin4-2* promoter from *Petroselinum crispum*; RB, right border; *Thsp*, terminator of *Arabidopsis heat shock protein 18.2*; *Tnos*, *nos* terminator; *TPab5*, terminator of *Arabidopsis polyadenylate-binding protein 5*.

Two vector constructs, pBIN_*PcUbi*_*Eluc*_*Thsp* and pBIN_*PcUbi*_*E-RS-luc*_*Thsp* (Figure 1), were designed for the experiment to monitor luminescence in *C. morifolium* plants. The *PcUbi* promoter is derived from the *ubiquitin4-2* promoter of *Petroselinum crispum* (Plesch and Edneth 2011) and was amplified from pBCKK-*PcUbi*pro::GUS-*HSP*T (Kishi-Kaboshi et al. 2019). The *Eluc* PCR product was amplified from the pELuc-test plasmid (Toyobo, http://www.toyobo-global.com/seihin/xr/lifescience/). The terminator of the *Arabidopsis heat shock protein 18.2* (*Thsp*, (Nagaya et al. 2010)) were derived from pRI201-AN (Takara). The *PcUbi* promoter, *Eluc*, and *Thsp* PCR products were assembled in the pBI121 vector by seamless cloning system, yielding pBIN_*PcUbi*_*Eluc*_*Thsp*.

The *EL-pTpab5nptIIb-UC* DNA fragment was used to generate pBIN_*PcUbi*_*E_RS_luc*_*Thsp*. At first, the cauliflower mosaic virus *35S* promoter (*P35S*) derived from pRI201-AN (Takara) and the *Thsp* PCR products were inserted into the AscI/EcoRI site of the pPZP2028 vector (Endo et al. 2016) by an infusion reaction (Takara), yielding pPZP_P35S_Thsp. The *pTpab5nptIIb* fragment was constituted by the terminator of *Arabidopsis* *polyadenylate-binding protein 5* (*Tpab5*), *nopaline synthase* (*nos*) promoter, *neomycin phosphotransferase* II (*npt* II), and *nos* terminator (*Tnos*). The fragment of tobacco mosaic virus 5′UTR Ω and *Eluc* gene with the *piggyBac* inverted-repeat transposable element (IVR) was digested with *Xba*I/*Sac*I from pE(L1-L2)Pef:ELpbUC:Thsp16.9 (Nishizawa-Yokoi et al. 2014), then, it was ligated between the *P35S* and *Thsp* of pPZP_P35S_Thsp, yielding pPZP_P35S_ELpbUC_Thsp. The DNA fragments of the *Tpab5* and *npt* II expression cassette [*nos* promoter: *npt* II: *Tnos*] were amplified and inserted into the *Avr*II/*Sal*I site within the *piggyBac* IVR of pPZP_P35S_*ELpbUC*_*Thsp*, yielding pPZP_P35S_*EL-pTpab5nptIIb-UC*_*Thsp*. The *EL-pTpab5nptIIb-UC_Thsp* and the *PcUbi* promoter DNA fragments were inserted into the HindIII/EcoRV site of pBI121 vector by seamless cloning system, yielding pBIN_*PcUbi*_*E_RS_luc*_*Thsp*. The inserted sequences of these vectors were confirmed by the Sanger sequencing method.

**Figure 2:**
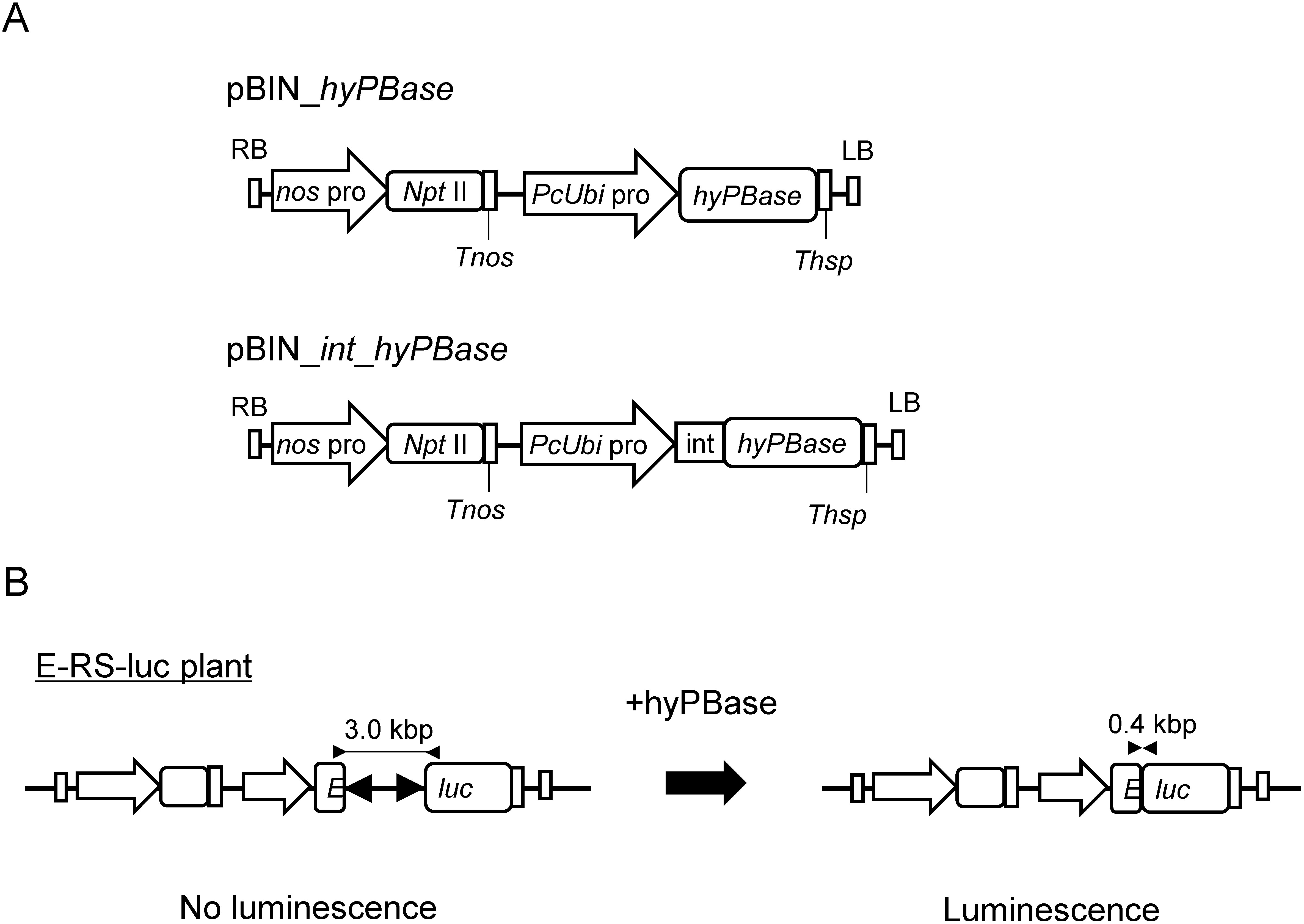
Figure 2. Structures of *hyPBase* expressing construction and scheme of the excision of a DNA sequence. A: Structures of the binary vectors to make transgenic *A. tumefaciens*. LB, left border; RB, right border; *PcUbi*, *ubiquitin4-2* promoter from *Petroselinum crispum*; *nos* pro, *nos* promoter, *npt* II; *neomycin phosphotransferase* for kanamycin resistance, *Tnos*, *nos* terminator; *hyPBase*, *hyperactive piggyBac transposase*; int, intron of caster bean *catalase*; *Thsp*, terminator of *Arabidopsis heat shock protein 18.2.* B: Scheme of the excision of a DNA sequence from the plant by hyPBase. In the E-RS-luc transgenic plant, the Eluc gene construct was divided by the RS region and inactive. When hyPBase excises the *RS* region, the Eluc gene construct becomes intact and active. The position of the primers used to amplify the E-RS-luc or Eluc DNA fragment is indicated by arrow heads.

Two vector constructs, pBIN_*hyPBase* and pBIN_*int_hyPBase* (Figure 2), were designed for express *hyPBase* in *C. morifolium* and generated as follows. The *NLS_FLAG_hyPBase* from pPN/hyPBase (Nishizawa-Yokoi et al. 2014), the *PcUbi* promoter, and the *Thsp* PCR products and assembled into the HindIII/EcoRI site of pBI121 vector by seamless cloning system, yielding pBIN*_hyPBase PcUbi* promoter. The *int_NLS_FLAG_hyPBase*_*Thsp* from pE(L1-L2)PcUbi_ADHintNLSflaghyPBase_Tathsp, the *PcUbi* promoter, and the *Thsp* PCR products were assembled into HindIII/EcoRV site of the pBI121 vector by seamless cloning system, yielding pBIN_*int_hyPBase*. pE(L1-L2)PcUbi_ADHintNLSflaghyPBase_Tathsp was constructed as follows. The fragment of the *PcUbi* promoter and *Thsp* was amplified with primers (Table 1) and was integrated into the AscI/PacI site of pENTR L1/L2 (Thermo Fisher), yielding pE(L1-L2)PcUbi:Tathsp.

**Table 1:** Table 1. Oligonucleotide sequences used in this study.

Target and purpose	Direction	Sequence
Generation of pBIN_*PcUbi*_*Eluc*_*Thsp* or pBIN_*PcUbi*_*E-RS-luc*_*Thsp*		
PcUbi	Forward	5′-TGGGAGGCCTGgagctctcaGAAACAGCTCTGGCA-3′
	Reverse	5′-TCTCTCCATCTGCACATACATAACATATCAAGA-3′
Eluc and E-RS-luc	Forward	5′-TATGTGCAGATGGAGAGAGAGAAGAACGTGG-3′
	Reverse	5′-CCTTAGTACTTTACAGCTTAGAAGCCTTCTCCA-3′
Thsp	Forward	5′-TAAGCTGTAAAGTACTAAGGGCGAATTCCAGC-3′
	Reverse	5′-TATGTGCAGATGGAGAGAGAGAAGAACGTGG-3′
Generation of pBIN_*int_hyPBase* or pBIN*_hyPBase*		
PcUbi	Forward	5′-ATGACCATGATTACGCCACTAGCAACGATTGTACAATTGCT-3′
	Reverse	5′-GGTTCCCATCTGCACATACATAACATATCAAGA-3′
int_NLS_FLAG_hyPBase_Thsp	Forward	5′-TATGTGCAGATGGGGTAAATTCTAGTTTTTCTCCT-3′
	Reverse	5′-GTAAAACGACGGCCAGTGCTTATCTTTAATCATATTCCATAGTCCA-3′
NLS_FLAG_hyPBase	Forward	5′-TATGTGCAGATGGGAACCAAGAAGAAGAGA-3′
	Reverse	5′-ATCTTCATATTCAGAAACAGCTCTGGCACA-3′
Thsp	Forward	5′-CTGTTTCTGAATATGAAGATGAAGATGAAATATTTGGTGT-3′
	Reverse	5′-GTAAAACGACGGCCAGTGCTTATCTTTAATCATATTCCATAGTCCA-3′
Construction of the pPZP_*P35S_EL-pTpab5nptIIb-UC*_Thsp vector		
Amplification of *Tpab5*		
pig-Tpab5 F	Forward	5′-AACTTTTAtcctaggacatactgtttactcaagac-3′
Tpab5-Pnos R	Reverse	5′-TCTCCGCTCATGATCcaagcaccaccgcgatttgg-3′
Amplification of the *npt* II cassette		
Tpab5-Pnos F	Forward	5′-tcgcggtggtgcttgGATCATGAGCGGAGAATTAA-3′
Tnos-pig R	Reverse	5′-CTgacgtccgtcgacAATTCCCGATCTAGTAACAT-3′
Construction of the pE(L1-L2)PcUbi_ADHintNLSflaghyPBase_Tathsp and pE(L1-L2)PcUbi:Tathsp vectors		
Amplification of PcUbi:Tathsp		
attL1-PcUbi F	Forward	5′-AAAAAAGCAGgctggcgcgccCTAGCAACGATTGT-3′
Tathsp_attL2-R	Reverse	5′-CTGggttaatcctgcaggCTTATCTTTAATCATATTCC-3′
Amplification of *hyPBase*		
hyPBase SpeI-F	Forward	5′-AGGTTAATGGAactagtGGCAGCAGCCTGGACGAC-3′
hyPBase SacI-R	Reverse	5′-TGGGAGGCCTGgagctctcaGAAACAGCTCTGGCA-3′
Amplification of PcUbi-*E-RS-luc_Thsp*		
PCR	Forward	5′-GTAAAACGACGGCCAG-3′
	Reverse	5′-CAGGAAACAGCTATGAC-3′
Amplification of *Eluc* or *E-RS-luc*		
PCR	Forward	5′-ACACTACTGTGAAGCTGGGC-3′
	Reverse	5′-CACAGGGTCGTAGTGCAGAG-3′
Sequence analysis		5′-CCGATCCTGGTCTTCACCAC-3′

The artificially synthesized 346 bp DNA fragment of the *Arabidopsis alcohol dehydrogenase* 5′UTR+*Castor bean catalase intron*+*Simian virus 40 NLS*+*FLAG tag* and the *hyPBase* fragment amplified by PCR were introduced into the *Xba*I/*Sac*I site of pE(L1-L2)PcUbi:Tathsp, yielding pE(L1-L2)PcUbi_ADHintNLSflaghyPBase_Tathsp. The pBIN_*int_hyPBase* contains an intron sequence from *catalase* in *Ricinus communis* before *NLS*, *FLAG* tag, and a *hyPBase* sequence, which is codon-optimized for eudicots. The *hyPBase* nucleotide sequences in pBIN*_hyPBase* and pBIN_*int_hyPBase* are almost identical except for one nucleotide between the *FLAG tag* and *hyPBase* sequences. There was no amino acid difference. The inserted sequences in these vectors were confirmed by the Sanger sequencing method. All oligonucleotide sequences used in this work are indicated in Table 1.

### Generation of transgenic plants

A *C. morifolium* cultivar, ‘Sei-Marin’ (Inochio Seikoen Inc., http://www.seikoen-kiku.co.jp/en/), was used as a transformation host. The binary vectors, pBIN_*PcUbi*_*Eluc*_*Thsp* and pBIN_*PcUbi*_*E-RS-luc*_*Thsp*, were introduced into the *Agrobacterium tumefaciens* strain EHA105 by electroporation. Transgenic plants were generated as previously described (Aida et al. 2004) without wash step after co-culture with *Agrobacterium*. Transgenic plants (Eluc and E-RS-luc) harboring binary vectors pBIN_*PcUbi*_*Eluc*_*Thsp* or pBIN_*PcUbi*_*E-RS-luc*_*Thsp* were vegetatively maintained on a half-strength Murashige and Skoog (MS) salt medium without hormones containing an MS vitamin, 3% sucrose, and 0.25% gellum gum under a standard light intensity (photon flux density 70 µmol s^−1^ m^−2^) at 20°C.

### Inoculation of *Agrobacterium* on Eluc or E-RS-luc transgenic plants

*Agrobacterium* inoculation was performed as usual *C. morifolium* transformation method (Aida et al. 2004) without wash step after co-culture with *Agrobacterium*. In brief, *Agrobacterium* was suspended in 10 mM MgSO_4_, 1% tween 20, and 100 µM acetosyringone at OD 600 nm 0.15 in NanoDrop One (Thermo Fisher Scientific, https://www.thermofisher.com/jp/ja/home.html). Leaf disks were immersed in the bacterial suspension for 20 min and transferred to a cocultivation medium (MS salt medium, MS vitamin source, 3% sucrose, 1 mg ml^−1^ 6-benzyl adenine, 2 mg ml^−1^ α-naphthalene acetic acid, 100 µM acetosyringone, and 0.1% gellan gum) and incubated at 20°C for 2 days in dark condition. For the mock treatment, 10 mM MgSO_4_, 1% tween 20, and 100 µM acetosyringone were used. The inoculated leaf disks were transferred to a selection medium (MS salt medium, MS vitamin source, 3% sucrose, 1 mg ml^−1^ 6-benzyl adenine, 0.5 mg ml^−1^ α-naphthalene acetic acid, 300 mg l^−1^ carbenicillin, and 0.8% agar), incubated at 20°C for 8/16 h in light/dark condition, and subcultured for every two weeks in fresh medium. The shoots emerged around three months post-inoculation, detached from the callus, and grown on half-strength MS salt medium without hormones and with an MS vitamin source, 3% sucrose, and 0.25% gellan gum at 20°C.

### Luciferin treatment and detection of luminescence

Whole leaf or transgenic calli were sprayed with D-luciferin solution (300 mg l^−1^ D-luciferin, 20 mM sodium phosphate, pH 7.0). The luminescence was visualized with an imaging system at Chemi-Hi Resolution mode and an exposure time of 120 min (Chemidoc MP and Image Lab 4.0 software, Bio-Rad, CA, USA).

### DNA analysis

Leaves from transgenic plants or calli from *Agrobacterium*-inoculated samples were collected and stored at −30°C. Genomic DNA was extracted using a Plant DNA Isolation Reagent (Takara clontech). We used PrimeStarGXL DNA polymerase (Takara clontech) to amplify the DNA. Short *Eluc* DNA fragments from Ag-hyPBase-inoculated E-RS-luc plants were excised from the agarose gel, purified with Nucleospin Gel and PCR Clean-up kit (Macherey-Nagel), and sequenced using a capillary DNA analyzer. All oligonucleotide sequences are indicated in Table 1.

## Results and discussion

In this study, we constructed the system which visualize the DNA excision event by luminescence. First, we generated the pBIN_*PcUbi_Eluc_Thsp* vector containing the *Emerald luciferase* (*Eluc*) gene between the parsley *ubiquitin4-2* (*PcUbi*) promoter and *Arabidopsis heat shock protein 18.2* terminator (*Thsp*) as a positive control (Figure 1A). Transgenic *C. morifolium* plants (Eluc plant) was generated using the pBIN_*PcUbi_Eluc_Thsp* vector. We obtained 20 Eluc plant lines, which were confirmed by PCR to possess the *Eluc* sequence. We evaluated luciferase activity in leaves from 14 transgenic Eluc plant lines. Five plant lines (#52, #72, #74, #78, and #79) showed clear luminescence, especially two of them (#74 and #79) showed strong luminescence after D-luciferin treatment (Figure 1B). This indicated that the PcUbi-Eluc-Thsp construct and D-luciferin treatment could be used to monitor Eluc activity in *C. morifolium*.

Next, we generated a pBIN_*PcUbi_E-RS-luc_Thsp* vector, which contained the E-RS-luc construct between the *PcUbi* promoter and the *Thsp*. The E-RS-luc construct have the Eluc sequence with the 2.6 kbp insert DNA, *pTpab5nptIIb* sequence (Figure 1C). The *Eluc* sequence in the *EL-pTpab5nptIIb-UC* sequence could not be translated as luciferase by insertion. When the *piggyBac* transposase excises DNA sandwiched between two *piggyBac* IVRs from the genome, the *Eluc* sequence was recovered and could be translated as luciferase. Transgenic plants of *C. morifolium* (E-RS-luc plants) were generated using the pBIN_*PcUbi_E-RS-luc_Thsp* vector. We obtained eight Eluc plant lines (#1, #4, #5, #8, #9, #10, #13, and #14), which were confirmed by PCR to possess 5.5 kbp entire PcUbi_E-RS-luc_Thsp sequence (Supplementary Figure S1).

We incorporated a *hyPBase* expression cassette on binary vectors to apply *piggyBac* transposase in *C. morifolium*. It is known that the transgene expression level in rice callus is increased when the first intron of the castor bean *catalase* gene was inserted in the transgene (Tanaka et al. 1990). We prepared two binary vectors, pBIN_*int_hyPBase* and pBIN*_hyPBase* (NLS, nuclear localization signal; Figure 2A, B), with or without intron sequence from the castor bean *catalase* gene just after the start codon of the *NLS-FLAG-hyPBase* open reading frame, with the expectancy of enhancement of transgene expression.

**Figure 3:**
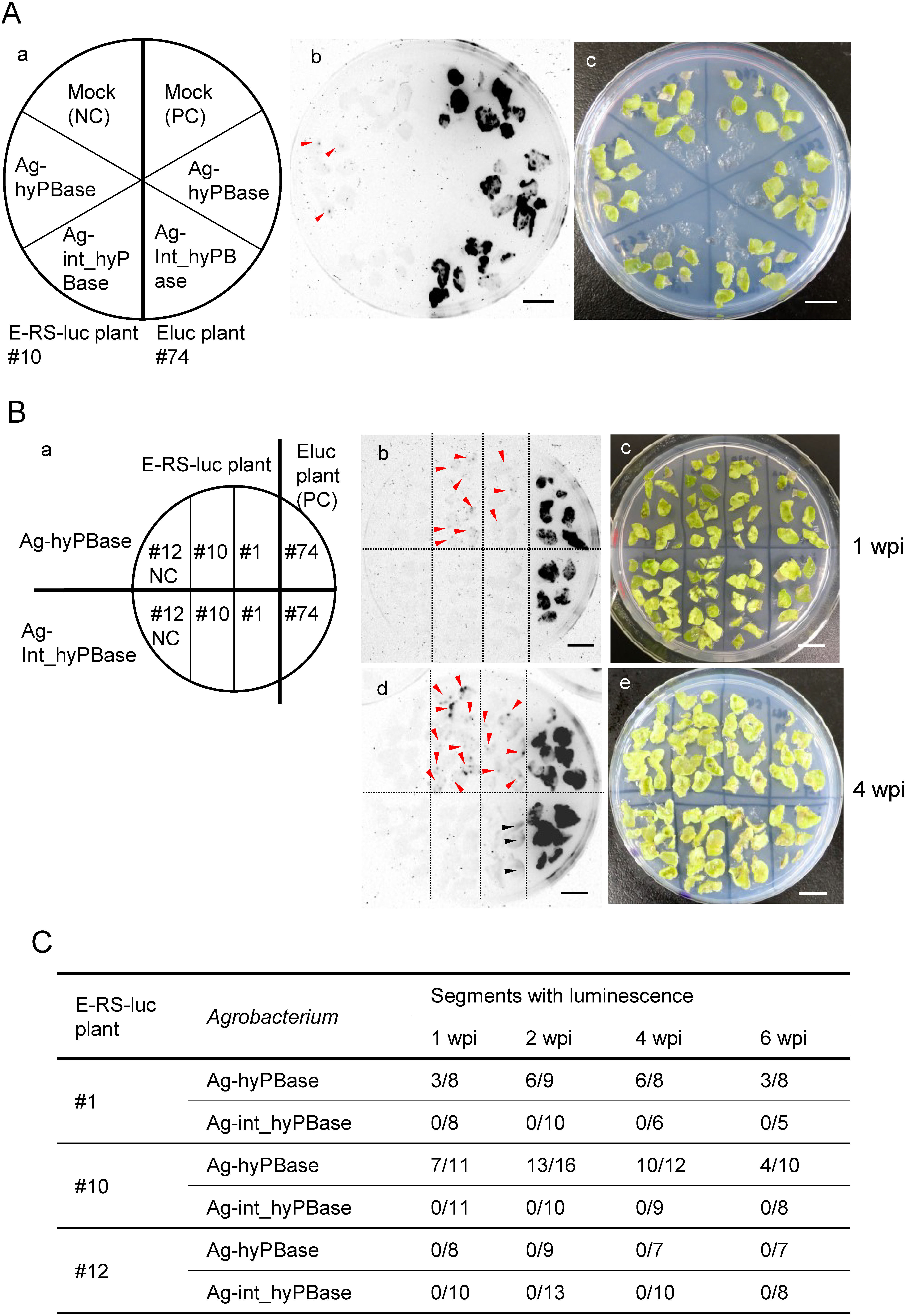
Figure 3. Luminescence in E-RS-luc plants inoculated with Ag-hyPBase. A: Luminescence of leaf segments of Eluc #74 and E-RS-luc #10 plants inoculated with mock, Ag-hyPBase, or Ag-int-hyPBase at two weeks post-inoculation. The left panel (a) indicates the position of each plant and the treatment. Luminescence (middle panel, b) and visible (right panel, c) images are shown. The position of the luminescent site in the E-RS-luc plant is indicated by red arrowheads. Bars indicate 1 cm length. B: Luminescence of Eluc #74 and E-RS-luc #1, #10, and #12 plants inoculated with Ag-hyPBase, or Ag-int-hyPBase at 1 (b and c) and 4 (d and e) weeks post-inoculation (wpi). The positions of each plant and the treatment are indicated in the left panel (a). Luminescence (middle panel, b and d) and visible (right panel, c and e) images are shown. The position of the luminescent site in the E-RS-luc plant is indicated by red arrowheads. The position of the accumulated luminescent outflow from Eluc plants is indicated by black arrowheads. Bars indicate 1 cm length. C: Number of leaf segments with luminescence spots per used leaf segment. PC, positive control; NC, negative control.

Leaf disks from the Eluc #74 and E-RS-luc #10 plants were inoculated with *A. tumefaciens* (Ag-hyPBase and Ag-int-hyPBase). Then, it was transformed with either the pBIN*_hyPBase* or the pBIN_*int_hyPBase* vector. The Ag-hyPBase-inoculated E-RS-luc #10 plants showed punctate luminescence sites in leaf disks. In contrast, mock-inoculated E-RS-luc plants did not show luminescence at two weeks post-inoculation (Figure 3A). The Ag-hyPBase-inoculated E-RS-luc #1 and #10 plants began to show a punctate luminescence site in the leaf disk one week after inoculation (Figure 3B). The E-RS-luc #12 plant was confirmed by PCR to contain truncated *E-RS-luc* sequence but not to have intact *PcUbi_E-RS-luc_Thsp* construct (Supplementary Figure S1) and used as a negative control for luminescence. The luminescence sites were more clearly visible in Ag-hyPBase-inoculated E-RS-luc #1 and #10 plants four weeks after inoculation. We counted the number of leaves with luminescence after *Agrobacterium* inoculation (Figure 3C). The E-RS-luc plants #1 and #10 consistently showed luminescence after inoculation with Ag-hyPBase. On the other hand, luminescence was not detected in the Ag-int-hyPBase-inoculated leaves of E-RS-luc plants.

**Figure 4:**
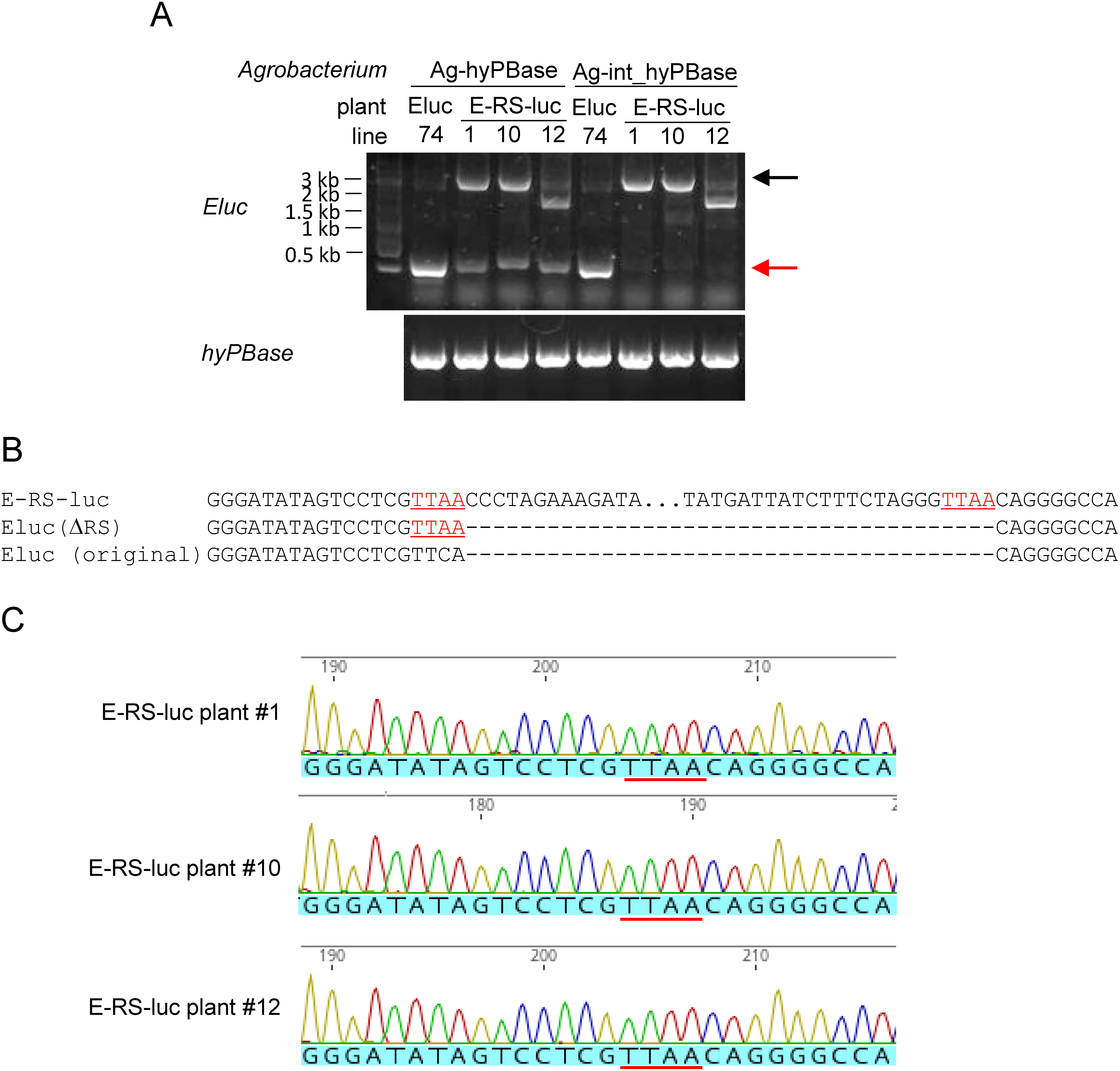
Figure 4. Eluc DNA fragments were excised from the E-RS-luc plant inoculated with Ag-hyPBase. A: Eluc and E-RS-luc size DNA fragments were detected in E-RS-luc plants inoculated with Ag-hyPBase at two weeks post-inoculation. The position of the E-RS-luc size DNA fragments is indicated by the black arrow. The position of the Eluc size DNA fragments is indicated by the red arrow. B: Alignment of DNA fragments surrounding RS insertion sites of Eluc and E-RS-luc fragments. The red color and under bar indicate the position of the TTAA site recognized by *piggyBac* transposase. C: Sanger sequencing of the PCR-amplified Eluc fragment from E-RS-luc plants inoculated with Ag-hyPBase. DNA bands were excised from the agarose gel and analyzed. The position of the TTAA site is indicated by the red line.

Next, we performed PCR analysis to amplify the Eluc or E-RS-luc sequences for the DNA extracted from leaf disks inoculated with Ag-hyPBase. A small fragment with electromobility similar to the Eluc plant was detected in Ag-hyPBase-inoculated E-RS-luc #1, #10, and #12 plants. In contrast, no such fragment was detected in the E-RS-luc plants inoculated with Ag-int-hyPBase (Figure 4A). This result was consistent with the luminescence of E-RS-luc leaf disks inoculated with Ag-hyPBase or Ag-int-hyPBase (Figure 3B). The DNA band of the PCR-amplified E-RS-luc sequence was faint in E-RS-luc #12 plant. A smaller DNA band than E-RS-luc PCR fragments were shown in Ag-hyPBase or Ag-int-hyPBase-inoculated samples (Figure 4A). This suggested that the E-RS-luc #12 plants had truncated T-DNA, which had the Eluc sequence but would lack the *PcUbi* promoter sequence, in its genome. After the Ag-hyPBase inoculation, the *RS* sequence was excised from the E-RS-luc #12 plant, but the recovered *Eluc* sequence might not be functional.

We recovered and sequenced the small DNA fragments of E-RS-luc plants inoculated with Ag-hyPBase from the agarose gel. The conjugated Eluc sequence after the excision of sequences sandwiched between *RS* sequences from E-RS-luc sequence is expected to differ from that in the Eluc plant (Figure 4B). Sequences from all E-RS-luc plants inoculated with Ag-hyPBase showed a TTAA site (Figure 4C) that did not exist in the Eluc plant. These results indicated that the DNA fragment was derived from *E-RS-luc* constructs and that the DNA fragment between two *piggyBac* *RS* sequences was excised from the *E-RS-luc* sequence. Despite our expectation that the castor bean *catalase* first intron would enhance transgene expression, inoculation of Ag-int-hyPBase did not result in excising DNA from the E-RS-luc plant. The intron-mediated enhancement was used to boost transgene expression in various plants, but the enhancement mechanism in monocot and dicot plants would be different (Laxa 2017). For *C. morifolium* leaf segments, the castor bean *catalase* first intron did not enhance the expression of *hyPBase*. It is also possible that the splicing of the inserted intron led irregular transcription of *hyPBase* gene. It might be possible that other intron sequences and/or other intron position enhance transgene expression in *C. morifolium*.

We tried to gain regenerated shoots from the E-RS-luc leaf segments, which have an RS-excised-Eluc sequence. We used 89 leaf segments and obtained one shoot from the E-RS-luc #10 plant inoculated with Ag-hyPBase in one experiment. We performed two independent experiments, but the regeneration rates were very low in both experiments. In addition, overgrowth of *Agrobacterium* often killed the regenerated shoot. Finally, we obtained one shoot from the E-RS-luc #1 plant, two from the E-RS-luc #10 plant inoculated with Ag-hyPBase, and four from the E-RS-luc #1 plant inoculated with Ag-int-hyPBase. However, none had a short DNA fragment by PCR analysis of the E-RS-luc sequence. It would be required to use more leaf segments to obtain the regenerated shoots with the RS-excised-Eluc sequence from the E-RS-luc leaf segments and to restrict growth of *Agrobacterium* in tissue culture.

In this study, we performed excision of DNA sequences from the *C. morifolium* genome using the *piggyBac* system. The occupancy of the luminescence site in inoculated leaf disks suggests that the frequency of DNA excision is not very high as expected. However, we can detect excision events on our usual transformation scale using 100–400 leaf disks. The leaf segments with luminescent spots were observed in more than 50% of the Ag-hyPBase-inoculated E-RS-luc leaves of plants #1 and #10. The regeneration efficiency from the E-RS-luc transgenic plant inoculated with Ag-hyPBase was not as good as the usual transformation process using non-transgenic *C. morifolium* (data not shown). A larger-scale experiment will yield DNA-excised plants using our E-RS-luc transgenic plant. We maintained transgenic *C. morifolium* plants vegetatively in a plant box and used them for analysis. Continuous cultivation under aseptic conditions and consecutive transformation processes might badly influence regeneration. We believe it is possible to generate a more efficient system to obtain DNA-excised shoots with the improvement of the *Agrobacterium* inoculation and regeneration conditions and the development of a more efficient *piggyBac* expression system with an intron enhancer in *C. morifolium* tissue culture. Otherwise, it would be possible to perform genome editing first and then remove the genome editing tool using *piggyBac* with an inducible expression system in one transformation event to avoid low regeneration efficiency.

We used approximately 1.8-kbp DNA fragments to evaluate hyPBase activity in *C. morifolium*. The length of *Cas9* from *Streptococcus pyogenes* (*SpCas9*) is 4.1 kbp. The lengths of commonly used plant promoters and terminators are as follows: 0.3 kbp for cauliflower mosaic virus *35S* promoter, 0.9 kbp for *PcUbi* promoter, 0.3 kbp for *nos* promoter, 0.3 kbp for *A. thaliana* *hsp* terminator, and 0.3 kbp for *nos* terminator. Therefore, a minimal length of approximately 5 kbp is required to express *SpCas9*. The original *piggyBac* element is 2.5 kbp (Cary et al. 1989). HyPBase has been demonstrated to excise 4.3-kbp DNA sequences in rice (Nishizawa-Yokoi et al. 2014). The *piggyBac* can transfer 14.3 kbp and 100 kbp DNA sequences in mouse cells (Ding et al. 2005; Li et al. 2011). Therefore, it will be possible to excise the *Cas9* expression cassette used for genome editing in *C. morifolium*. We believe that the excision of a genome editing tool from genome-edited *C. morifolium* will be one of the solutions applied to generate mutant plants in *C. morifolium* by genome editing for commercial use.

## Abbreviations

Cas9: CRISPR-associated protein 9
CRISPR: clustered regularly interspaced short palindromic repeats
Eluc: emerald luciferase
hyPBase: hyperactive *piggyBac* transposase
IVR: inverted-repeat transposable element
MS: Murashige and Skoog
NLS: nuclear localization signal
*nos*: *nopaline synthase*
*npt* II: *neomycin phosphotransferase* II
*P35S*: cauliflower mosaic virus *35S* promoter
*PcUbi*: *Petroselinum crispum* *ubiquitin4-2*
RS: recognition site
*SpCas9*: *Cas9* from *Streptococcus pyogenes*
*Thsp*: terminator of *Arabidopsis* *heat shock protein 18.2*
*Tpab5*: terminator of *Arabidopsis* *polyadenylate-binding protein 5*
