# Supplementary material for: Excision of DNA fragments with the piggyBac system in Chrysanthemum morifolium
Source: Plant Biotechnol (Tokyo). 2023 Jun 25;40(2):157–65. doi: 10.5511/plantbiotechnology.23.0324a (PMC10797517; doi:10.5511/plantbiotechnology.23.0324a)
Supplement: Supplementary Data [file plantbiotechnology-40-2-23.0324a-s001.pdf]

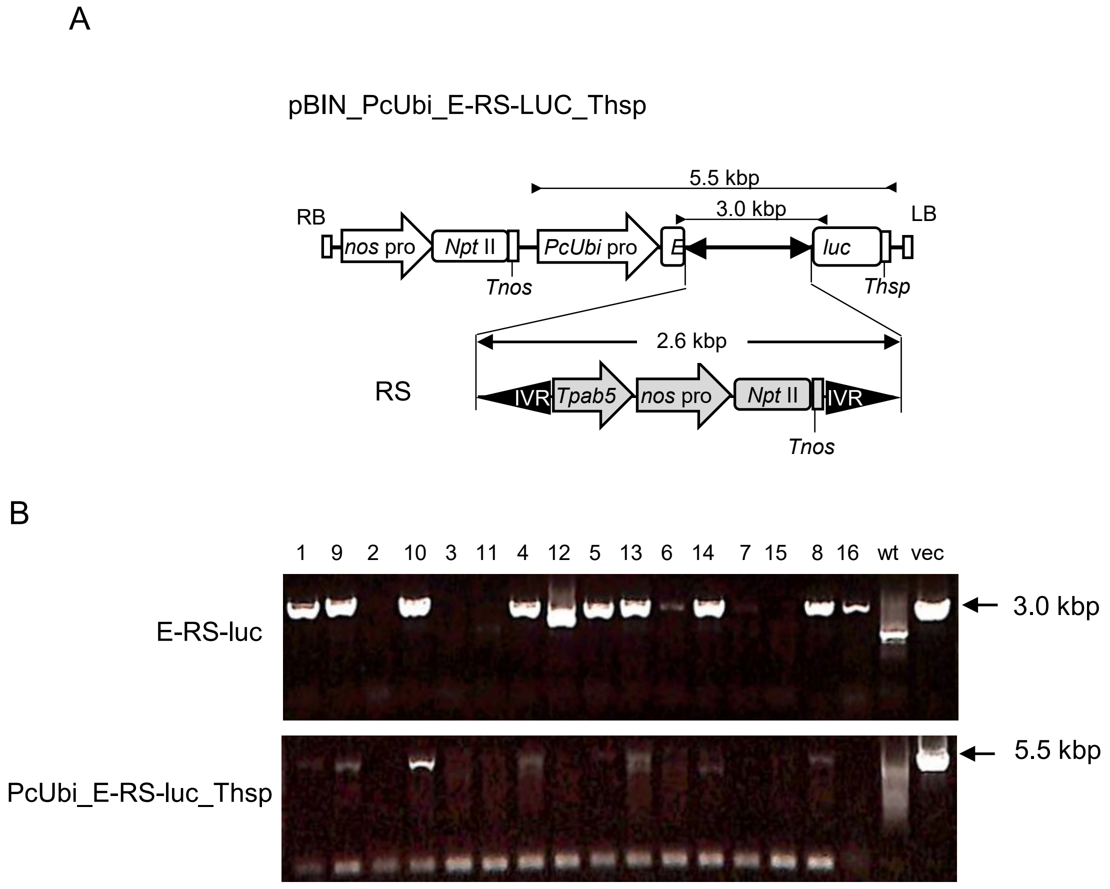

Supplemental Figure 1. Selection of transgenic E-RS-luc plant.

A. Structures of pBIN_*PcUbi_E-RS-luc_Thsp* to generate transgenic E-RS-luc plants. The position of primers used to amplify DNA fragments is indicated with arrow heads. *Eluc*, *emerald luciferase*; IVR, inverted repeat of *piggyBac* transposon; LB, left border; *nos* pro, *nos* promoter, *Npt* II; *neomycin phosphotransferase* for kanamycin resistance, *PcUbi*, *Ubiquitin4-2* promoter from *Petroselinum crispum*; RB, right border; *Thsp*, terminator of *Arabidopsis heat shock protein 18.2*; *Tnos*, *nos* terminator; *Tpab5*, terminator of *Arabidopsis polyadenylate-binding protein 5.*

B. Gel image of partial E-RS-luc (upper panel) and entire PcUbi_E-RS-luc_Thsp (lower panel) DNA fragments. The position of E-RS-luc and PcUbi_E-RS-luc_Thsp DNA fragments is indicated with arrows. vec, pBIN_*PcUbi_E-RS-luc_Thsp* binary vector used as positive control of PCR analysis; wt, DNA from no transgenic *C. morifolium* plant used as negative control of PCR analysis.
